# Chronic Intermittent Hypobaric Hypoxia Decreases High Blood Pressure by Stabilizing the Vascular Renin-Angiotensin System in Spontaneously Hypertensive Rats

**DOI:** 10.3389/fphys.2021.639454

**Published:** 2021-03-24

**Authors:** Hua Chen, Bin Yu, Xinqi Guo, Hong Hua, Fang Cui, Yue Guan, Yanming Tian, Xiangjian Zhang, Yi Zhang, Huijie Ma

**Affiliations:** ^1^Department of Physiology, Hebei Medical University, Shijiazhuang, China; ^2^Department of Cardiovascular Care Unit, Hebei General Hospital, Shijiazhuang, China; ^3^Department of Emergency, Fourth Hospital of Hebei Medical University, Shijiazhuang, China; ^4^Department of Electron Microscope Experimental Centre, Hebei Medical University, Shijiazhuang, China; ^5^Hebei Collaborative Innovation Center for Cardio-cerebrovascular Disease, Shijiazhuang, China

**Keywords:** chronic intermittent hypobaric hypoxia, anti-hypertension, endothelial-dependent relaxation, renin-angiotensin system, Ang II, Ang1-7, AT1 receptor, Mas receptor

## Abstract

**Background and Aims:**

Previous studies have demonstrated the anti-hypertensive effect of chronic intermittent hypobaric hypoxia (CIHH) in hypertensive rats. The present study investigated the anti-hypertensive effect of CIHH in spontaneously hypertensive rats (SHR) and the role of the renin-angiotensin system (RAS) in anti-hypertensive effect of CIHH.

**Methods:**

Fifteen-week-old male SHR and WKY rats were divided into four groups: the SHR without CIHH treatment (SHR-CON), the SHR with CIHH treatment (SHR-CIHH), the WKY without CIHH treatment (WKY-CON), and the WKY with CIHH treatment (WKY-CIHH) groups. The SHR-CIHH and WKY-CIHH rats underwent 35-days of hypobaric hypoxia simulating an altitude of 4,000 m, 5 h per day. Arterial blood pressure and heart rate were recorded by biotelemetry, and angiotensin (Ang) II, Ang1–7, interleukin (IL)-6, tumor necrosis factor-alpha (TNF)-α, and IL-10 in serum and the mesenteric arteries were measured by enzyme-linked immunosorbent assay (ELISA) and immunohistochemistry, respectively. The microvessel tension recording technique was used to determine the contraction and relaxation of the mesenteric arteries. Hematoxylin and eosin and Masson’s staining were used to observe vascular morphology and fibrosis. Western blot was employed to detect the expression of the angiotensin-converting enzyme (ACE), ACE2, AT1, and Mas proteins in the mesenteric artery.

**Results:**

The biotelemetry result showed that CIHH decreased arterial blood pressure in SHR for 3–4 weeks (*P* < 0.01). The ELISA and immunohistochemistry results showed that CIHH decreased Ang II, but increased Ang1–7 in serum and the mesenteric arteries of SHR. In the CIHH-treated SHR, IL-6 and TNF-α decreased in serum and the mesenteric arteries, and IL-10 increased in serum (*P* < 0.05–0.01). The microvessel tension results revealed that CIHH inhibited vascular contraction with decreased Ang1–7 in the mesenteric arteries of SHR (*P* < 0.05–0.01). The staining results revealed that CIHH significantly improved vascular remodeling and fibrosis in SHR. The western blot results demonstrated that CIHH upregulated expression of the ACE2 and Mas proteins, and downregulated expression of the ACE and AT1 proteins (*P* < 0.05–0.01).

**Conclusion:**

CIHH decreased high blood pressure in SHR, possibly by inhibiting RAS activity, downregulating the ACE-Ang II-AT1 axis and upregulating the ACE2-(Ang1-7)-Mas axis, which resulted in antagonized vascular remodeling and fibrosis, reduced inflammation, and enhanced vascular relaxation.

## Introduction

Hypertension is a common cardiovascular disease and the main risk factor for malignant cardiovascular events, such as coronary heart disease and stroke. The incidence of hypertension is increasing worldwide, and it has become a killer of human health. Primary hypertension accounts for about 90% of hypertension cases, and although progress has been made in the study of the mechanism of primary hypertension in recent years, the cause and pathogenesis of primary hypertension remain unclear (Rossier et al., 2017; Reboussin et al., 2018).

Numerous studies have demonstrated the beneficial effects of chronic intermittent hypobaric hypoxia (CIHH) on the heart, brain, liver, and kidneys under ischemia/reperfusion (I/R) or pathological conditions (Zhang and Zhou, 2012; Yuan et al., 2015; Tian et al., 2016; Zhang et al., 2016). Our previous studies showed that CIHH protects the heart against I/R injury, promotes the recovery of cardiac function, reduces the myocardial infarct area, and antagonizes arrhythmias in the I/R heart (Zhang et al., 2000; Zhou et al., 2013). In addition, CIHH effectively reduces arterial blood pressure in renovascular hypertensive rats, which may be related to the facilitation of the baroreflex and enhanced vascular relaxation (Guan et al., 2016; Li et al., 2016).

The renin-angiotensin system (RAS) is an important humoral regulatory system in the body. The RAS regulates cardiovascular activity and maintains the water and electrolyte balance and internal environmental homeostasis. However, the abnormal activation of the RAS results in hypertension under pathological conditions (Li et al., 2017). Two opposite and mutually restricted functional axes have been demonstrated in the RAS: the angiotensin-converting enzyme (ACE)-angiotensin (Ang) II-AT1 receptor axis and the ACE2-(Ang 1–7)-Mas receptor axis. The balance of these axes is an important guarantee of the steady-state of the RAS (Nehme et al., 2019). In addition to the circulating RAS, another independent RAS, called the local RAS, plays a more direct and important role in regulating cardiovascular activity than the circulating RAS (Agoudemos and Greene, 2005; Littlejohn et al., 2016). It is unknown whether the anti-hypertensive effect of CIHH is related to inhibiting the RAS.

Spontaneously hypertensive rats (SHRs) are an ideal animal model of hereditary hypertension to simulate human primary hypertension and are widely used (Pravenec et al., 2000). Some studies have shown that CIHH treatment can reduce the blood pressure of SHRs (Serebrovskaya et al., 2008), but the mechanism is unclear.

Thus, we hypothesized that CIHH would have an anti-hypertensive effect by inhibiting the RAS, downregulating the ACE-Ang II-AT1 receptor axis, and upregulating the ACE2-(Ang 1–7)-Mas receptor axis to enhance vascular relaxation. In this study, biotelemetry, microvessel tension recording, enzyme-linked immunosorbent assay (ELISA), and immunohistochemical and molecular biology methods were employed to investigate the antihypertensive effects of CIHH and the underlying mechanism in SHRs.

## Materials and Methods

### Animal Groups and CIHH Treatment

All experiments were carried out in compliance with the Guide for the Care and Use of Laboratory Animals as adopted and promulgated by the U.S. National Institutes of Health, and they were reviewed and approved by the Ethics Committee for the Use of Experimental Animals at Hebei Medical University (IACUC-Hebmu-2018005).

Forty-six SHRs (15-weeks-old) and thirty Wistar-Kyoto (WKY) rats of the same age were purchased from the Beijing Vital River Laboratory Animal Technology Co. (Beijing, China). The rats were divided into the SHR control group (SHR-CON), the SHR with CIHH treatment group (SHR-CIHH), the WKY control group (WKY-CON), and the WKY and CIHH treatment group (WKY-CIHH). SHR-CIHH and WKY-CIHH rats were placed in a hypobaric chamber to receive a 35-day hypobaric hypoxia treatment simulating an altitude of 4,000 m for 5 h per day. The CIHH-treated animals went through a 4-day adaptation before the regular CIHH treatment as follows: hypobaric hypoxia treatment simulating 1,000-m altitude for 1 h, 2,000-m for 2 h, 3,000-m for 3 h, and 4,000-m for 4 h, respectively. The CIHH treatment was always performed at a fixed time (8:00 am–1:00 pm) each day. All animals were housed in a temperature-controlled room (22 ± 1°C) under a 12 h/12 h light/dark cycle and had free access to water and food. The animal’s health state and physical activity were monitored every day, and body weight was measured once weekly.

### Blood Pressure (BP) Measurements With Biotelemetry

Surgery was performed aseptically in WKY rats and SHRs anaesthetized with 2–3% isoflurane. A catheter attached to a transmitter (Data Sciences International, St. Paul, MN, United States) was inserted into the abdominal aorta for BP monitoring and the transmitter body was implanted in the abdominal cavity. The rats were housed individually and were inspected daily for motor activity, signs of infection, and food and water intake. Arterial blood pressure and heart rate (HR) were measured and analyzed with a data acquisition system (Data Sciences International) in freely moving rats after recovery.

### Blood Biochemical Assays

Blood samples from WKY rats and SHRs were collected from the caudal vein the day after the *in vivo* experiment. The blood samples were centrifuged at 3,500 rpm for 10 min to recover serum for assay. ELISAs were used to measure serum Ang II, Ang1–7, IL-6, tumor necrosis factor (TNF)-α, and interleukin (IL)-10 (Elabscience Biotechnology Co., Ltd., Wuhan, China). The standard or sample was added to each well of the micro ELISA plate. The Biotinylated Detection antibody was added and incubated for 45 min at 37°C. Then the HRP conjugate was incubated, followed by the substrate reagent and stop solution. Then the plate was read immediately at an absorbance of 450 nm using a microplate reader.

### Vascular Ring Contraction and Relaxation Measurement

Control and CIHH-treated rats were euthanized with an intraperitoneally injected over-dose of pentobarbital sodium. The second-order mesenteric arteries were dissected and cut into 2-mm segments in Krebs–Henseleit (K-H) solution consisting of (mmol/L) NaCl 118.0, KCl 4.7, CaCl_2_ 2.5, MgSO_4_ 1.2, NaHCO_3_ 25.0, KH_2_PO_4_ 1.2, and glucose 11.0, pH 7.4. The arterial rings were mounted on 40-μm wires in a myograph system (Danish Myotech Technology, Aarhus, Denmark) and pre-incubated in 5 mL of oxygenated (95% O_2_, 5% CO_2_) K-H solution at 37°C, pH7.4, and then normalized and stabilized for 30 min. The isolated vessels were normalized using the DMT Normalization Module, which was used to calculate and set the optimal pre-tension conditions for the microvessels. The normalization procedure determined the internal circumference at which the vessel was under an optimal pre-tension and a transmural pressure of 100 mmHg. This index was denoted IC100 and was calculated for each vessel mounted on the wire myograph. The resulting force exerted by the vessel walls was recorded in LabChart. More information on the normalization of microvessels can be found in studies by Professor Michael Mulvany (Mulvany and Halpern, 1977).

The endothelium is considered intact if the vasorelaxation induced by acetylcholine (Ach, 10^–5^ M) in a vessel exceeds 70% of the contraction induced by phenylephrine (PE, 10^–5^ M). Vessel rings with an intact endothelium were induced to contract with KCl (20 mM, Sigma, St. Louis, MO, United States) and PE (10^–5^ M, Sigma), respectively, and relaxation of the vessel rings was induced with nine serially diluted concentrations of Ach (10^–9^–10^–5^ M, Sigma). In a separate set of experiments, the arterial rings were treated with five serially diluted concentrations of Ang II (10^–8^–10^–6^ M, Sigma) to test the Ang II-induced contraction in the different groups. In another separate set of experiments, Ang1–7 (10^–9^–10^–5^ M, BACHEM, Torrance, CA, United States) and ACh (10^–9^–10^–5^ M) were administered to test the vessel relaxation precontracted with PE (10^–5^ M). Vascular relaxation was expressed as a percentage of maximum steady-state contraction tension induced by PE (10^–5^ M) (Cui et al., 2018).

### Hematoxylin and Eosin (HE) Staining and Masson’s Staining

Based on a previous study, the 4% buffered formalin-fixed vessel tissues were embedded in paraffin (Grant et al., 1993). Tissue sections of 4-μm thickness were prepared. After removing the paraffin from the samples, they were stained with HE and observed and captured using a light microscope and an attached camera. The wall thickness and perimeter of the vessels were measured using an image processing system (Motic Med 6.0, Xiamen, China). The diameter of the vessels was calculated by using the mathematical equation (Diameter = Perimeter/π).

The samples for Masson staining were fixed in 4% formalin for 24 h at room temperature, permeabilized in xylene, embedded in paraffin, and then sliced into 4-μm-thick sections with a microtome. The sections were stained with 0.7% Masson-Ponceau-acid fuchsin staining solution and 2% aniline blue dye solution. Images of the stained sections were observed and captured with a light microscope and a camera. Collagen deposition (blue area) was semi-quantitatively analyzed with Image-Pro Plus software. The collagen volume fraction was calculated as the ratio of the collagen area to the entire tissue area of the visual field. A minimum of five randomly selected areas per sample was observed at ×200 magnification, and the average value was calculated for the statistical analysis.

### Immunohistochemistry

Immunohistochemistry was used to detect the expression of Ang II, Ang1–7, IL-6, TNF-α, and IL-10 in the mesenteric arteries. The samples were fixed in 4% formalin for 24 h at room temperature, permeabilized with xylene, embedded in paraffin, and then sliced into 4-μm-thick sections with a microtome. The sections were incubated with the primary antibodies, including rabbit anti-rat Ang II (#bs-0587R, 1:100, Beijing Biosynthesis Biotechnology Co.), rabbit anti-rat Ang 1–7 (#bs-20101R, 1:100, Beijing Biosynthesis Biotechnology Co.), rabbit anti-rat IL-6 (#bs-1782R, 1:300, Beijing Biosynthesis Biotechnology Co.), rabbit anti-rat TNF-α (#17590-1-AP, 1:300, Proteintech), and goat anti-rat IL-10 (#20850-1-AP, 1:300, Proteintech), respectively. After incubation with the secondary antibodies, DAB-substrate was incubated for 5 min at room temperature. Images of the stained sections were observed and captured with a light microscope and a camera. The positive areas were semi-quantitatively analyzed with Image Pro Plus software.

### Western Blotting

The expression levels of ACE, ACE2, the AT1 receptor, and the Mas receptor in the mesenteric arteries were measured by western blot. Proteins from the vessel tissues were extracted with a RIPA lysis/extraction buffer in the presence of a protease and phosphatase inhibitor cocktail, and the protein concentration was determined with a BCA kit (Beyotime). The samples (40 μg) were subjected to 4–15% Tris–HCl sodium dodecyl sulfate-polyacrylamide gel electrophoresis and transferred to a polyvinylidene difluoride membrane. The membranes were incubated with rabbit monoclonal anti-ACE antibody (ACE: #ET1705-36, 1:1,000, HuaAn Biotechnology Co. Ltd., Hangzhou, China), rabbit monoclonal ACE2 (#ET1611-58 1:2,000, HuaAn Biotechnology Co. Ltd.), rabbit polyclonal anti-angiotensin-(1–7) Mas Receptor antibody (#TA328708, 1:1,000, OriGene Technologies Inc., Rockville, MD, United States), rabbit polyclonal anti-AGTR1 antibody (AT-1, #A14201, 1:1,000 AB Clonal Biotechnology, Woburn, MA, United States), and rabbit monoclonal β-actin antibody (#20536-1-AP, 1:10,000, Proteintech) overnight at 4°C. The western blot analyses were quantified with Image J software.

### Statistical Analysis

All data were expressed as mean ± standard error. Statistical analysis was conducted using a two-way analysis of variance (ANOVA) followed by a Turkey’s *post-hoc* test for comparison among multiple groups of different concentrations and times. One-way ANOVA followed by a Turkey’s *post-hoc* test were used to compare multiple groups. The *t*-test was used to compare two groups. A *P* < 0.05 was considered significant. The data analysis was carried out using GraphPad Prism 8 (GraphPad Software Inc.,La Jolla, CA, United States).

## Results

### Effect of CIHH on Body Weight and Arterial Pressure

**FIGURE 1:**
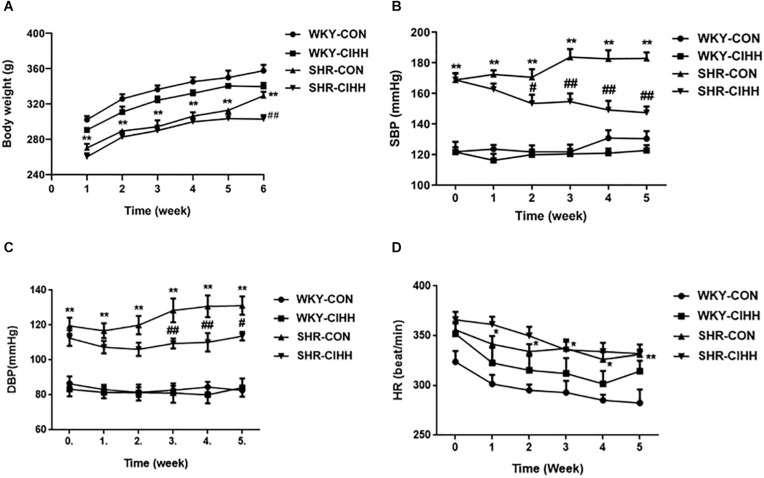
Effect of chronic intermittent hypobaric hypoxia (CIHH) on body weight and arterial blood pressure in spontaneously hypertensive rat (SHR). **(A)** Body weight; **(B)** systolic blood pressure (SBP); **(C)** diastolic blood pressure (DBP); **(D)** heart rate (HR). Data are expressed as mean ± SEM; SHR-CON: control SHR (*n* = 12); SHR-CIHH: SHR with CIHH (*n* = 12); WKY-CON: control WKY rats (*n* = 6); WKY-CIHH: WKY rats with CIHH (*n* = 6); *P < 0.05, **P < 0.01 vs. WKY-CON, ^#^*P* < 0.05, ^##^*P* < 0.01 vs. SHR-CON (two-way ANOVA).

Body weight decreased significantly in the SHR-CON rats compared with the WKY-CON rats before the CIHH treatment (*P* < 0.01). All rats gained weight during the 5-week CIHH treatment. However, body weight was lower in SHR-CIHH rats than that in SHR-CON rats at the end of the CIHH treatment (*P* < 0.05, Figure 1A), suggesting that CIHH reduces body weight.

**FIGURE 2:**
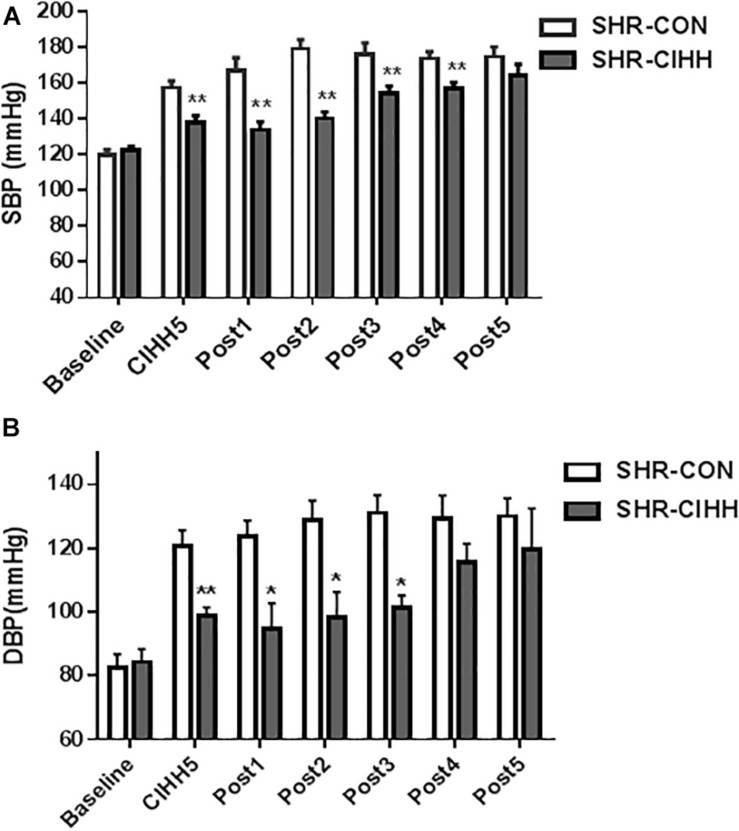
The duration of the depressor effect by chronic intermittent hypobaric hypoxia (CIHH) on SBP **(A)** and DBP **(B)** in spontaneously hypertensive rats (SHRs). **(A)** SBP: systolic blood pressure; **(B)** DBP: diastolic blood pressure. Data are expressed as mean ± SEM; SHR-CON: control SHR (*n* = 6); SHR-CIHH: SHR with CIHH (*n* = 6); CIHH5: 5 weeks of CIHH; Post 1–Post 5: 1–5 weeks after CIHH; *P < 0.05, **P < 0.01 vs. SHR-CON (*t*-test).

Arterial blood pressure, including systolic blood pressure (SBP) and diastolic blood pressure (DBP), increased significantly in SHR-CON rats compared with WKY-CON rats (*P* < 0.05–0.01), but decreased significantly in SHR-CIHH rats compared with SHR-CON rats (*P* < 0.01). HR increased significantly in SHR-CON rats compared with WKY-CON rats (*P* < 0.05), but CIHH did not affect HR of the WKY or SHR rats (*P* > 0.05, Figures 1B–D). The decreasing effect of CIHH on SBP and DBP in SHR lasted 3–4 weeks (Figure 2). These results indicate that CIHH had an effective anti-hypertensive effect but no effect on HR in hypertensive rats.

### Effect of CIHH on Ang II, Ang1–7, and Inflammation-Related Cytokines in Serum

**FIGURE 3:**
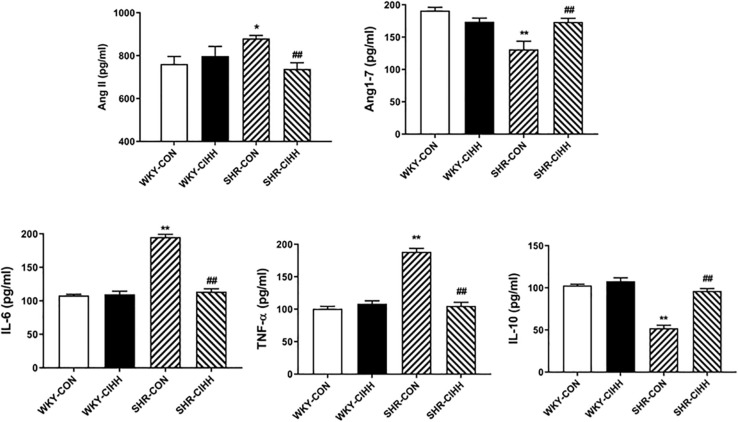
Effect of chronic intermittent hypobaric hypoxia (CIHH) on serum angiotensin II (Ang II), angiotensin 1–7 (Ang1–7), IL-6, TNFα, and IL-10 in spontaneously hypertensive rats (SHRs). Data are expressed as mean ± SEM; SHR-CON: control SHR (*n* = 14); SHR-CIHH: SHR with CIHH (*n* = 14); WKY-CON: control WKY rat (*n* = 6); WKY-CIHH: WKY rat with CIHH (*n* = 6); *P < 0.05, **P < 0.01 vs. WKY-CON ^##^*P* < 0.01 vs. SHR-CON (one-way ANOVA).

The ELISA results showed that the levels of serum Ang II, IL-6, and TNF-α increased significantly, whereas the levels of Ang 1–7 and IL-10 decreased significantly in SHR-CON rats compared with WKY-CON rats (*P* < 0.05–0.01). The serum levels of Ang II, IL-6, and TNF-α decreased, but the serum levels of Ang 1–7 and IL-10 increased in SHR-CIHH rats compared with SHR-CON rats (*P* < 0.01, Figure 3). These results indicate that the CIHH treatment prevented the increase in serum Ang II and pro-inflammatory cytokines and the decrease in serum Ang 1–7 and anti-inflammatory cytokine in hypertensive animals.

### Effect of CIHH on Ang II, Ang 1–7, and Inflammation-Related Cytokines in Vessels

**FIGURE 4:**
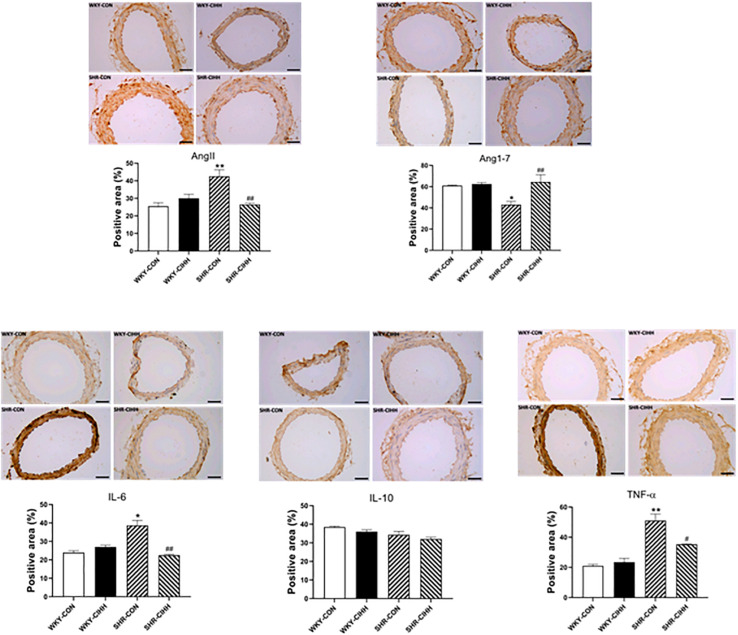
Effect of chronic intermittent hypobaric hypoxia (CIHH) on angiotensin II (Ang II), angiotensin 1–7 (Ang1–7), IL-6, TNF-α, and IL-10 in mesenteric arteries of spontaneously hypertensive rats (SHRs). Data are expressed as mean ± SEM; *n* = 5 for each group. SHR-CON: control SHR; SHR-CIHH: SHR with CIHH; WKY-CON: control WKY rats; WKY-CIHH: WKY rats with CIHH; *P < 0.05, **P < 0.01 vs. WKY-CON, ^#^*P* < 0.05, ^##^*P* < 0.01 vs. SHR-CON (one-way ANOVA).

The Immunohistochemistry results showed a significant increase in the positive areas expressing Ang II (*P* < 0.01) and a decrease in the positive areas expressing Ang1–7 (*P* < 0.05) in the mesenteric arteries of the SHR-CON rats compared with the WKY-CON rats. Also the expression levels of IL-6 and TNF-α (*P* < 0.01) increased significantly in the SHR-CON rats. The positive areas expressing Ang II decreased significantly in the mesenteric arteries of SHR-CIHH rats compared with those in SHR-CON rats (*P* < 0.01), but the positive areas expressing Ang1–7 increased significantly (*P* < 0.01). In addition, IL-6 (*P* < 0.01) and TNF-α (*P* < 0.05, Figure 4) expression decreased significantly in SHR-CIHH rats. These results indicate that the CIHH treatment opposed the changes in Ang II, Ang1–7, IL-6, and TNF-α in the mesenteric arteries of hypertensive rats.

### Effect of CIHH on Vascular Ring Contraction

#### KCl-Induced Contraction

**FIGURE 5:**
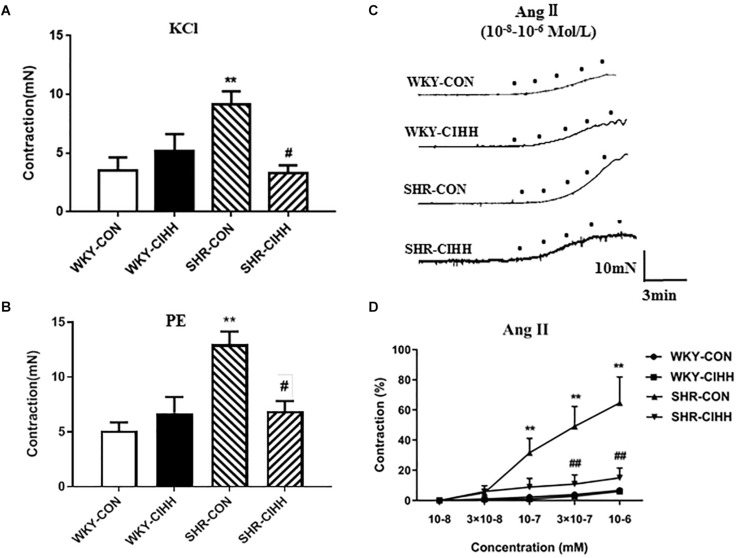
Effect of chronic intermittent hypobaric hypoxia (CIHH) on PE-induced, KCl-induced, and Ang II-induced contraction of the mesenteric arteries from spontaneously hypertensive rats (SHRs). Summarized data of mesenteric arteries in response to KCl **(A)** and PE **(B)**. Representative contraction traces **(C)** and summarized data **(D)** of mesenteric arteries in response to different concentrations of Ang II. Data are expressed as mean ± SEM; SHR-CON: control SHR (*n* = 14); SHR-CIHH: SHR with CIHH (*n* = 12); WKY-CON: control WKY rats (*n* = 12); WKY-CIHH: WKY rats with CIHH (*n* = 12); **P < 0.01 vs. WKY-CON, ^#^*P* < 0.05, ^##^*P* < 0.01 vs. SHR-CON [one-way ANOVA for **(A,B)**, two-way ANOVA for **(C)**].

KCl-induced contraction in mesenteric arteries increased significantly in SHR-CON rats compared with WKY-CON rats (*P* < 0.01) but decreased significantly in SHR-CIHH rats compared with SHR-CON rats (*P* < 0.05, Figure 5A).

#### PE-Induced Contraction

PE-induced contraction was significantly enhanced in mesenteric arteries of SHR-CON rats compared with those of WKY-CON rats (*P* < 0.01) but decreased significantly in SHR-CIHH rats compared with SHR-CON rats (*P* < 0.05, Figure 5B).

#### Ang II-Induced Contraction

Different concentrations of Ang II(10^–8^ M, 3 × 10^–8^ M, 10^–7^ M, 3 × 10^–7^ M, 10^–6^ M)were applied to understand the response of mesenteric arteries to Ang II. The contraction tension of the vessels increased along with the increase of Ang II. Ang II-induced contraction was significantly stronger in SHR-CON rats than that in WKY-CON rats (*P* < 0.01), and the concentration-response curve shifted left and upward. However, Ang II-induced contraction was significantly lower in SHR-CIHH rat than that in SHR-CON rats (*P* < 0.01), and the concentration-respond curve shifted right and downward (Figure 5C).

These results indicate that the CIHH treatment significantly inhibited the enhancement of contraction induced by KCl, PE, and Ang II, and antagonized the increase in contractive reactivity of Ang II in mesenteric arteries of hypertensive rats.

### Effect of CIHH on the Relaxation of Vascular Rings

#### ACh-Induced Endothelial-Dependent Relaxation

**FIGURE 6:**
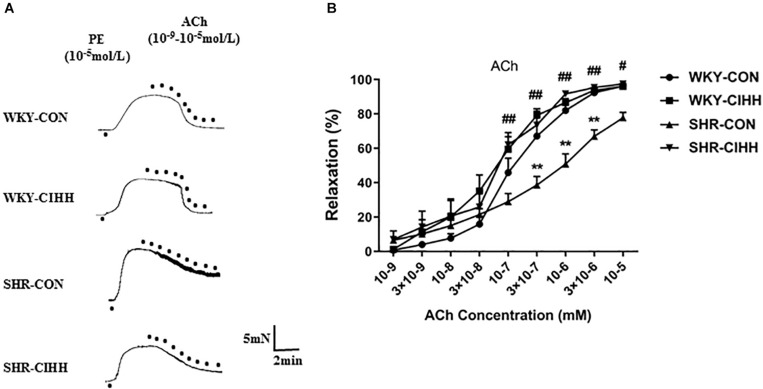
Effect of chronic intermittent hypobaric hypoxia (CIHH) on ACh-induced relaxation in the mesenteric arteries of spontaneously hypertensive rats (SHRs). Representative traces **(A)** and summarized data **(B)** of mesenteric arteries in response to different concentrations of ACh after precontracted with PE. Data are expressed as mean ± SEM; SHR-CON: control SHR (*n* = 16); SHR-CIHH: SHR with CIHH (*n* = 14); WKY-CON: control WKY rats (*n* = 12); WKY-CIHH: WKY rats with CIHH (*n* = 12). **P < 0.01 vs. WKY-CON, ^#^*P* < 0.05, ^##^*P* < 0.01 vs. SHR-CON (two-way ANOVA).

Different concentrations of ACh (10^–9^–10^–5^ M) were used to understand the effects of CIHH on ACh-induced endothelial-dependent relaxation. Vascular relaxation increased gradually in each group along with the increase of ACh concentration. ACh-induced relaxation was significantly weaker in SHR-CON rats than that in WKY-CON rats, and the concentration-response curve shifted right and downward (*P* < 0.01). While ACh-induced relaxation was significantly stronger in SHR-CIHH rats than that in SHR-CON rats, the concentration-response curve shifted left and upward (*P* < 0.05–0.01, Figure 6). These results indicate that the CIHH treatment enhanced endothelial-dependent relaxation and reversed the decrease in endothelial-dependent relaxation in hypertensive rats.

#### Ang1-7-Induced Relaxation

**FIGURE 7:**
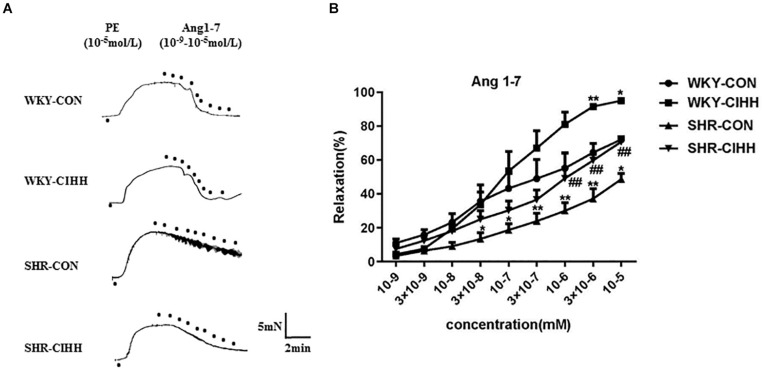
Effect of chronic intermittent hypobaric hypoxia (CIHH) on Ang 1–7-induced relaxation in mesenteric artery of spontaneously hypertensive rat (SHR). Representative traces **(A)** and summarized data **(B)** of mesenteric arteries in response to different concentrations of Ang 1–7 after precontracted with PE. Data are expressed as mean ± SEM; SHR-CON: control SHR (*n* = 16); SHR-CIHH: SHR with CIHH (*n* = 14); WKY-CON: control WKY rats (*n* = 12); WKY-CIHH: WKY rats with CIHH (*n* = 12). *P < 0.05, **P < 0.01 vs. WKY-CON, ^##^*P* < 0.01 vs. SHR-CON (two-way ANOVA).

Different concentrations of Ang1–7 (10^–9^–10^–5^ mM) were used to understand the effect of CIHH on Ang1–7-induced relaxation. Vascular relaxation increased gradually in each group along with the increase of Ang1-7 concentration. In SHR-CON rats, Ang1–7-induced relaxation was significantly weaker than that in WKY-CON rats, and the concentration-response curve shifted right and downward (*P* < 0.05–0.01). Ang1–7-induced relaxation was significantly greater in SHR-CIHH rats than in SHR-CON rats, and the concentration-response curve shifted left and upward (*P* < 0.01, Figure 7). These results indicate that the CIHH treatment enhanced endothelial-dependent relaxation and reversed the weakness of Ang1–7-induced relaxation in hypertensive rats.

### Effect of CIHH on Arterial Morphosis and Fibrosis

**FIGURE 8:**
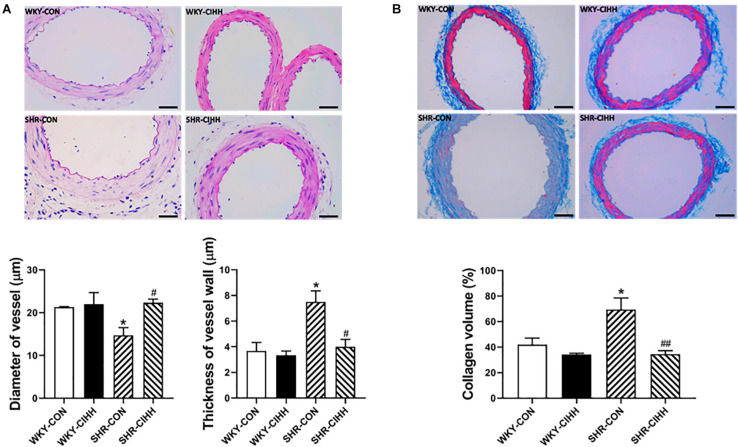
Effect of chronic intermittent hypobaric hypoxia (CIHH) on vascular structure and collagen volume in the mesenteric arteries of spontaneously hypertensive rats (SHRs). **(A)** Representative HE staining and summarized vessel diameter and vessel wall thickness data in the different groups: Scale bar 50 μm, magnification × 400, **(B)** representative and summarized Masson’s staining data: Scale bar 50 μm, magnification × 400, Data are expressed as mean ± SEM; *n* = 5 for each group. SHR-CON: control SHR; SHR-CIHH: SHR with CIHH; WKY-CON: control WKY rats; WKY-CIHH: WKY rats with CIHH. *P < 0.05 vs. WKY-CON, ^#^*P* < 0.05, ^##^*P* < 0.01 vs. SHR-CON (one-way ANOVA).

The HE staining results revealed significant morphological changes in the mesenteric arteries from SHR-CON rats compared with those from WKY-CON rats, as manifested by a narrow tube cavity and thickened tube wall. The CIHH treatment significantly improved these changes in hypertensive rats. The quantified data also show that the increased diameter of the vessels and the thickness of vessel walls improved after the CIHH treatment in SHRs (*P* < 0.05, Figure 8A).

Masson’s staining was used to detect collagen fibers in micro-arteries, and the relative collagen content value is represented by the collagen volume fraction. The results showed that the collagen content in the mesenteric arteries and the volume fraction of collagen increased significantly (*P* < 0.05). The CIHH treatment decreased the collagen content and the collagen volume fraction in the mesenteric arteries of hypertensive rats (*P* < 0.01, Figure 8B).

### Effect of CIHH on the Expression of ACE, ACE2, AT1, and Mas

**FIGURE 9:**
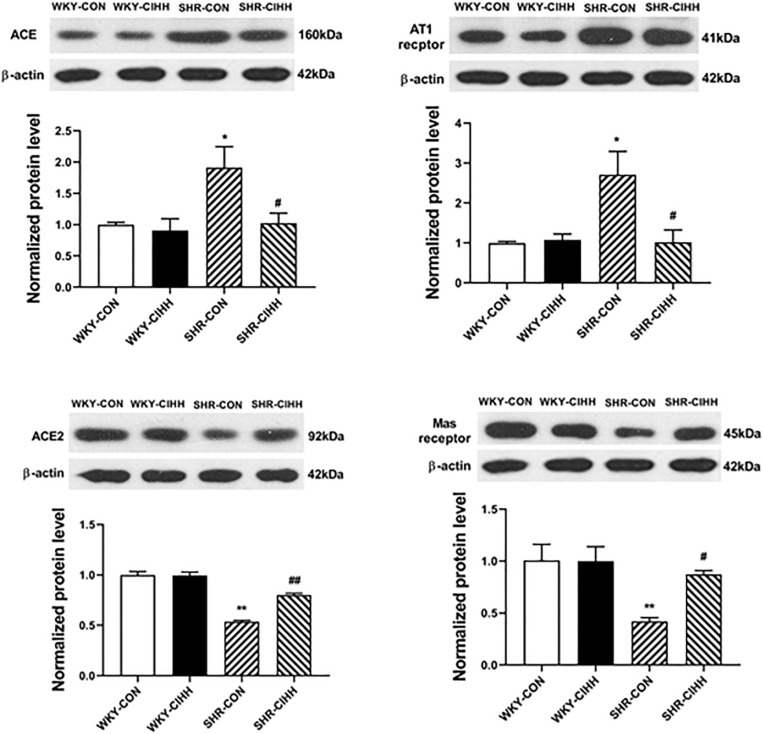
Effect of chronic intermittent hypobaric hypoxia (CIHH) on the expression of ACE, ACE2, AT1, and Mas in the mesenteric arteries of spontaneously hypertensive rats (SHRs). Data are expressed as mean ± SEM; *n* = 6 for each group. SHR-CON: control SHR; SHR-CIHH: SHR with CIHH; WKY-CON: control WKY rats; WKY-CIHH: WKY rats with CIHH; *P < 0.05, **P < 0.01 vs. WKY-CON, ^#^*P* < 0.05, ^##^*P* < 0.01 vs. SHR-CON (one-way ANOVA).

The western blot results showed that ACE and AT1 expressions were upregulated but that expressions of ACE2 and Mas were downregulated in the mesenteric arteries of SHR-CON rats compared with those in WKY-CON rats (*P* < 0.05–0.01). ACE and AT1 expressions were downregulated, whereas ACE2 and Mas expressions were upregulated in the mesenteric arteries of SHR-CIHH rats compared with SHR-CON rats (*P* < 0.05–0.01, Figure 9). These results indicate that the CIHH treatment antagonized the upregulation of ACE and AT1 and downregulation of ACE2 and Mas in the mesenteric arteries of hypertensive rats.

## Discussion

In the present study, we investigated the effect of CIHH on arterial blood pressure in SHRs. The results showed that CIHH effectively decreased the high blood pressure in SHRs, which lasted 3–4 weeks. Also, CIHH diminished the contraction but enhanced the relaxation of the mesenteric arteries. In addition, CIHH alleviated the remodeling, fibrosis, and inflammation in mesenteric arteries. Furthermore, the ACE- Ang II-AT1 axis was downregulated and ACE2-(Ang 1–7)-Mas axis was upregulated, suggesting that the anti-hypertensive effect of CIHH is related to the amelioration of vasomotion, inflammation, and RAS activity.

Peripheral resistance is a fundamental factor for the maintenance of normal arterial blood pressure under physiological conditions and is an important pathophysiological basis for hypertension (Rossier et al., 2017). The stability of peripheral resistance depends on the vascular morphological structure and vasomotor function. A large number of studies have shown that vascular contraction is increased and vascular relaxation decreased in patients with hypertension and hypertensive animals, such as SHR (Lee et al., 1991; Rizzoni et al., 1994; le Noble et al., 1998). Some studies have shown that changes in vascular structure, such as remodeling and fibrosis, are an earlier sign than increased blood pressure, suggesting that vascular remodeling and fibrosis also play an important role in the occurrence and development of hypertension (Park et al., 2019). Our previous studies on renovascular hypertensive rats showed that CIHH reduces blood pressure by enhancing endothelium-dependent and non-endothelium-dependent relaxation of the mesenteric arteries (Guan et al., 2016). In this study, the vascular remodeling and fibrosis were significantly alleviated, vessel contraction induced by KCl, PE, and Ang II decreased significantly, and vessel relaxation induced by ACh and Ang 1–7 was increased significantly in CIHH-treated SHRs, which may be a mechanism underlying the anti-hypertensive effect of CIHH.

Numerous recent studies have shown that hypertension is a chronic inflammatory disease, and that vascular inflammation and inflammation-related factors play an important role in the pathophysiological process of hypertension (Liang et al., 2017; Luong et al., 2018; Alanazi and Clark, 2019). Increased levels of IL-6 and TNF-α are detectable in multiple types of hypertension, such as obese hypertension, alcohol-induced hypertension, drug-induced diabetic hypertension, as well as correlative animal models of hypertension, which indicates that inflammatory cytokines such as IL-6 and TNF-α are involved in causing hypertension (Liang et al., 2017; Alanazi and Clark, 2019). Consistent with our previous study that CIHH inhibits the inflammatory response in collagen-induced arthritis rats (Shi et al., 2015), the present study further confirmed the inhibitory effect of CIHH on vascular inflammation in SHRs. Therefore, it is reasonable to assume that the anti-hypertensive effect of CIHH is at least partly due to its anti-inflammatory effects.

It is well-known that the RAS, an important humoral regulatory system, plays an important role in regulating cardiovascular activities. Under physiological conditions, the ACE-Ang II-AT1 axis and ACE2-(Ang 1–7)-Mas axis act opposite to each other, restricting each other and maintaining the normal functioning of RAS (Simões e Silva and Teixeira, 2016; Wang et al., 2016). Under pathological conditions, the ACE-Ang II-AT1 axis becomes over-activated, and RAS activity increases, leading to hypertension, atherosclerosis, hypertrophy, type 2 diabetes, and kidney fibrosis. Ang II combined with the AT1 receptor induces cellular hyperplasia, vascular remodeling and fibrosis, the inflammatory response, oxidative stress, and vessel contraction, leading to increased blood pressure (Ribeiro-Oliveira et al., 2008; Nehme and Zibara, 2017a,b). In contrast, the ACE2- (Ang 1–7)- Mas axis has a protective effect on cardiovascular activities by antagonizing the ACE-Ang II-AT1 axis. Ang 1–7 combined with the Mas receptor has anti-inflammation, anti-fibrosis, antioxidation, and vessel relaxation effects, resulting in decreased blood pressure (Li et al., 2017; Wang et al., 2017; Liao et al., 2019). In this study, the ACE-Ang II-AT1 axis was upregulated, whereas the ACE2-(Ang1–7)-Mas axis was downregulated in mesenteric arteries of CIHH-treated SHRs. Therefore, this study is the first to confirm that CIHH stabilizes RAS activity by downregulating the ACE-Ang II-AT1 axis and upregulating the ACE2-(Ang1–7)-Mas axis, consequently resulting in anti-inflammatory, anti-remodeling, anti-fibrosis, enhanced vasorelaxation, and reduced vasoconstriction.

Drug therapy remains a major treatment for hypertension. However, there is no ideal drug to cure hypertension completely although many new drugs have been developed. The adverse effect of these drugs is a clinical problem that cannot be ignored. Studies from ours and other laboratories have shown the anti-hypertension effects of CIHH in different hypertension animal models, such as renovascular hypertensive rats and SHRs (Katiukhin et al., 1979; Behm et al., 1984; Serebrovskaya et al., 2008; Li et al., 2019). Clinical studies have shown that CIHH, also called hypobaric intermittent hypoxia training, has a depressor effect in primary hypertensive patients and postmenopausal women with hypertension (Serebrovskaya et al., 2008; Tin’kov et al., 2011), indicating the universality of the CIHH anti-hypertension effect. In addition, CIHH treatment is a simple inexpensive operation with no obvious side effects and is expected to become a non-drug-assisted prevention and treatment method for hypertension.

A limitation of this study is that no causal relationship was established regarding the precise mechanism of the CIHH anti-hypertensive effect. This study clearly showed that CIHH downregulated the ACE- Ang II-AT1 axis, upregulated the ACE2- (Ang 1–7)- Mas axis, reduced vascular remodeling and fibrosis, improved inflammation, decreased vascular contraction and increased vascular relaxation, and lowered arterial blood pressure but did not provide direct evidence for an interaction between them. Further studies are needed to provide a more detailed mechanism for the CIHH anti-hypertensive effects using some drug tools, such as Mas receptor or AT1 receptor blockers.

## Conclusion

In conclusion, CIHH decreased high blood pressure in SHRs, which may have occurred by inhibiting RAS activity, downregulating the ACE-Ang II-AT1 receptor axis, and upregulating the ACE2-(Ang1–7)-Mas receptor axis. These processes antagonized vascular remodeling and fibrosis, reduced inflammation, and enhanced vascular relaxation.

## Data Availability Statement

The raw data supporting the conclusions of this article will be made available by the authors, without undue reservation.

## Ethics Statement

The animal study was reviewed and approved by the Ethics Committee for the Use of Experimental Animals in Hebei Medical University.

## Author Contributions

HM and YZ designed the experiments. HC, BY, XG, HH, FC, and YG performed the experiments. YT and YG analyzed the data. XZ prepared the images. HC wrote the manuscript. HM and YZ revised the manuscript. All authors contributed to the article and approved the submitted version.

## Conflict of Interest

The authors declare that the research was conducted in the absence of any commercial or financial relationships that could be construed as a potential conflict of interest.
